# Assessing mentalization in practice: Reliability of the mentalization‐based treatment research adherence and competence scale

**DOI:** 10.1111/bjc.70010

**Published:** 2025-08-10

**Authors:** Karen Yirmiya, Sophie Marjoribanks, Peter Fonagy, Anthony Bateman

**Affiliations:** ^1^ Research Department of Clinical, Educational and Health Psychology University College London London UK; ^2^ Anna Freud National Centre for Children and Families London UK; ^3^ Department of Psychology Ben‐Gurion University Be'er Sheva Israel

**Keywords:** adherence, competence, inter‐rater reliability, MBT‐RACS, mentalization‐based treatment, psychotherapy research, treatment fidelity

## Abstract

**Objectives:**

Mentalization‐Based Treatment (MBT) requires rigorous fidelity assessment to ensure accurate delivery and validate treatment efficacy. This study introduces the Mentalization‐Based Treatment Research Adherence and Competence Scale (MBT‐RACS), a new instrument developed initially for research purposes to align with contemporary MBT principles and address psychometric and conceptual limitations found in earlier adherence assessment approaches.

**Methods:**

Inter‐rater reliability of the MBT‐RACS was evaluated using 126 recorded MBT sessions (104 group, 22 individual), rated by 17 trained coders.

**Results:**

The results indicated strong overall reliability, with most domains demonstrating good to excellent inter‐rater agreement across both group and individual sessions, irrespective of ratings from two or three raters. Total adherence intraclass correlation coefficients (ICCs) were notably high for both group (.84) and individual (.95) sessions rated by two coders, substantially exceeding the reliability typically reported for comparable adherence instruments.

**Conclusions:**

These findings suggest that the MBT‐RACS's format, which emphasizes broader, clinically meaningful domains, may contribute to improved consistency in ratings. The scale's robust reliability supports its applicability in research and clinical supervision, enhancing methodological rigour, quality assurance and targeted feedback for effective MBT training and implementation.

**Practitioner points**

- The Mentalization‐Based Treatment Research Adherence and Competence Scale (MBT‐RACS) provides a highly reliable tool for evaluating therapist fidelity in both individual and group MBT sessions.
- Clinicians and supervisors with limited additional training can confidently use the MBT‐RACS to assess adherence and competence with as few as two raters, making quality assurance and targeted feedback more feasible in routine practice.
- The scale's strong reliability, particularly for the total adherence score, provides robust support for quality assurance in both clinical and research contexts.

## INTRODUCTION

Mentalization‐Based Treatment (MBT) is a psychotherapeutic approach aimed at strengthening the capacity to understand oneself and others in terms of internal mental states—such as thoughts, feelings, desires and beliefs. This capacity, known as mentalization or reflective functioning, is essential for effective self‐regulation and interpersonal functioning (Fonagy et al., 2018). MBT was initially developed for individuals with borderline personality disorder (BPD), who typically exhibit pronounced deficits in mentalizing (Fonagy & Bateman, 2006; Fonagy & Luyten, 2009; Vogt & Norman, 2019). However, its utility extends to a range of psychological disorders characterized by disruptions in social cognition, including antisocial personality disorder (ASPD), eating disorders, depression, self‐harm and attachment‐related difficulties (Bateman et al., 2016; Byrne et al., 2020; Fonagy et al., 2025; Hajek Gross et al., 2024; Malda‐Castillo et al., 2019; Sharp & Rossouw, 2024). By restoring and enhancing mentalizing capacities, MBT seeks to promote greater emotional resilience and relational stability across clinical populations (Fonagy et al., 2019).

In psychotherapy research, rigorous evaluation of treatment fidelity is essential, requiring precise assessment of therapists' adherence to and competence in delivering prescribed therapeutic techniques. Fidelity represents the extent to which an intervention is implemented exactly as intended (Bellg et al., 2004; Ginsburg et al., 2021), extending beyond mere delivery to encompass the quality and skill underlying therapeutic execution (Ginsburg et al., 2021). High adherence and competence underpin treatment integrity and significantly contribute to enhanced patient outcomes (Páez et al., 2025). Robust fidelity assessment allows clear differentiation of specific therapies from alternative interventions, ensuring observed outcomes can reliably be attributed to the intended treatment. This strengthens both internal validity—by maintaining consistency of the independent variable—and external validity, facilitating effective implementation across diverse contexts (Bellg et al., 2004; Bond & Drake, 2020). Conversely, inadequate fidelity or its poor measurement can lead to inaccurate inferences, complicating determination of whether observed effects reflect the intervention itself or deviations from the intended protocol (Bellg et al., 2004; Moncher & Prinz, 1991).

The Mentalization‐Based Treatment Adherence and Competence Scale (MBT‐ACS) was first introduced in 2013 to systematically evaluate MBT fidelity (Karterud et al., 2013). This scale comprised 17 defined clinician intervention items; each rated independently for adherence (frequency/occurrence) and competence (quality). Initial evaluation of the scale supported its utility but highlighted psychometric limitations. Karterud et al. (2013) reported high inter‐rater reliability for overall scores when multiple raters underwent extensive training; however, reliability decreased to moderate levels in more typical scenarios involving only two independent raters. Thus, achieving excellent reliability with the MBT‐ACS often required numerous raters or extensive training. Furthermore, item‐level analyses demonstrated considerable variability: some items attained good reliability, while others showed low or very low reliability. For example, the ‘stop and rewind’ intervention, a technique used to restart a mentalizing dialogue, was rarely observed in practice, limiting opportunities for consistent scoring. In contrast, interventions targeting ‘pretend mode’ may have occurred more frequently but proved difficult to code reliably, as raters often disagreed on which specific interventions qualified. This likely stemmed from limited operational clarity in the MBT‐ACS descriptions, making such theoretically important interventions challenging to apply and rate consistently. Such limitations underscored the need for a new approach to assessing adherence, prompting the development of an instrument designed specifically for research contexts and updated to reflect contemporary MBT principles.

Nevertheless, despite psychometric limitations, the MBT‐ACS demonstrated considerable utility, highlighting the essential role of fidelity tools as MBT expands into new clinical conditions and cultural contexts. The scale has been employed across adult populations with BPD in both individual and group sessions (Folmo et al., 2017; Kvarstein et al., 2015, 2019; Philips et al., 2018), including studies demonstrating its teachability and reliability in routine clinical settings (Simonsen et al., 2019), and among adolescents with conduct disorder (Hauschild et al., 2023). Additionally, the MBT‐CAS (Bate & Midgley, 2020) demonstrated validity for children in psychodynamic therapy (Güvenç & Halfon, 2023) and online psychotherapy formats (Coşkun et al., 2024). This broad applicability underscores the importance of ongoing, rigorous psychometric validation of MBT adherence measures across diverse contexts and populations, supporting its foundational role in MBT's global expansion.

Since the development of MBT adherence tools such as the MBT‐ACS, MBT‐AQS and MBT‐G‐AQS (Folmo et al., 2017; Karterud et al., 2013; Simonsen et al., 2019), MBT itself has evolved, notably with the publication of a comprehensive new clinical manual and supporting empirical studies offering updated guidelines for the implementation of individual and group MBT (Bateman et al., 2023; Bateman & Fonagy, 2016; Fonagy et al., 2025). In light of these developments, a new MBT Research Adherence and Competence Scale (MBT‐RACS) was created to align with the updated treatment model and to support systematic evaluation of therapist practice in research settings (Anna Freud National Centre for Children and Families, 2020). The MBT‐RACS maintains a core emphasis on therapist fidelity to MBT principles while introducing a restructured format and an expanded framework specifically designed for use in clinical trials. The scale is intended to facilitate quality assurance, provide targeted training feedback and enable detailed investigation into mechanisms of therapeutic change.

It highlights a central ‘not‐knowing stance’ maintained by the therapist across the session, includes a meta‐level rating of the overall structure constructed to facilitate mentalizing throughout the session and organizes the therapist's interventions into four core content domains: mentalizing process, identification of non‐mentalizing modes, mentalizing the affective narrative and relational mentalizing. Within each domain, specific example interventions (e.g. clarifying thoughts, handling pretend mode, exploring emotional context and significant interpersonal events, working with relationships) are noted by the rater as evidence, but scoring occurs at the domain level rather than for numerous discrete items. The session's final adherence and competence score comprises the aggregate of these domain scores together with stance and structure ratings. This new scale format aims to simplify the rating procedure, improve inter‐rater consistency by focusing on broader, clinically meaningful domains and ensure applicability across both individual and group therapy sessions. Indeed, the new scale was explicitly designed for use in individual MBT and MBT group therapy, including specific guidance to accommodate group‐session dynamics (e.g. employing a group ‘go‐around*’* to set the agenda as a structural element in group MBT). An international team of MBT experts contributed to the development of the MBT‐RACS, and preliminary use in clinical trials suggests it may prove to be a valuable assessment tool (Fonagy et al., 2025). However, a formal psychometric evaluation remains necessary.

The present study is the first to report empirical findings regarding the newly developed MBT‐RACS (Anna Freud National Centre for Children and Families, 2020). The primary objective was to evaluate the inter‐rater reliability of this scale and to assess whether it could be used consistently by multiple raters across two MBT delivery modalities: individual therapy sessions and group therapy sessions. By employing several trained coders to rate a substantial sample of MBT sessions, we aimed to evaluate inter‐rater agreement for each MBT‐RACS domain and the total scale scores in both individual and group settings. We hypothesized that this evaluation would formally establish the scale's suitability for future MBT research and training applications.

## METHODS

### Sample and raters

This study analysed 126 video‐recorded MBT sessions drawn from ongoing clinical programmes and trials. Of these, 104 were group therapy sessions (MBT‐G), each involving 4–6 patients typically facilitated by two co‐therapists, and 22 were individual therapy sessions involving one therapist and one patient. All sessions adhered to standard MBT protocols, with individual sessions lasting approximately 50 min and group sessions lasting approximately 75 min. Sessions were video‐recorded and retrospectively coded offline.

Seventeen trained raters (psychologists, psychiatrists or researchers experienced with MBT) participated in coding. All raters completed a one‐day (approximately 7‐h) MBT‐RACS training workshop, delivered in‐person by a senior MBT supervisor (AB). The training included didactic instruction on the new scale, detailed discussion of each domain and guided rating of pilot session recordings. Raters were required to rate 2 practice tapes without discussion and then reach preliminary agreement on these before participating in study ratings. The 17 raters included a mix of professionals with substantial MBT experience: 10 (59%) were certified MBT therapists actively delivering treatment, while the remainder were clinical researchers with prior experience using the original MBT‐ACS or involved in MBT trials. All raters had completed at least basic MBT clinical training and were familiar with the therapeutic model. This background was considered essential to ensure accurate domain‐level coding and interpretive judgement.

The approximate time required to rate a single session was 65 min for individual sessions and 120 min for group sessions, depending on session complexity. Only sessions rated by at least three raters were included. When sessions were coded by more than three raters, three raters were randomly selected for analyses to maintain consistency. Raters were blinded to each other's scores and, for research‐based sessions, blinded to patient outcomes. No raters evaluated sessions they themselves conducted. The video‐recorded MBT sessions were drawn from research studies and clinical quality monitoring procedures, all of which had received ethical approval from their respective institutional review boards. For participants involved in research trials, informed consent was obtained prior to their participation and the recording of therapy sessions.

### The mentalization‐based treatment research adherence and competence scale (MBT‐RACS)

The MBT‐RACS evaluates several domains of therapist behaviour, assessing fidelity to the MBT model. The scale includes two overarching domains: (1) the therapist's core mentalizing attitude, termed the *not‐knowing stance*, maintained throughout the session and (2) a meta‐domain rating the session's overall mentalizing structure. Additionally, the scale comprises four primary content domains: mentalizing process, identification of non‐mentalizing modes, mentalizing the affective narrative and relational mentalizing. Within each domain, raters evaluate the frequency and extensiveness of relevant intervention techniques. Each domain receives a rating on a 1–7 Likert scale based on these observations, with higher scores indicating greater adherence and competence.

A separate quality modifier (ranging from −1 to +1) is applied to the raw domain score based on the skilfulness of intervention delivery (e.g. timing, contextual appropriateness, sensitivity to alliance). In cases where a domain was clinically indicated but either absent or inadequately addressed, an absence penalty is applied. This ranges from −1 (indicating a clear failure to address a relevant domain) to +.5, which is awarded when the therapist's omission of the domain is judged to reflect skilful clinical restraint. For example, when intervening directly in a sensitive relational dynamic might have disrupted mentalizing or trust. The +.5 thus acknowledges thoughtful avoidance consistent with MBT principles. The final session score is calculated by summing adjusted domain scores for all rated domains, dividing by the number of rated domains and applying any absence penalties. An adherence rating between 3.5 and 4.5 is considered acceptable. This overall adherence and competence score represents a quantitative indicator of the therapist's fidelity to the MBT model for the assessed session.

### Statistical analysis

Descriptive statistics and inter‐rater reliability estimates were computed independently for each MBT‐RACS domain and the total adherence score, separately for group and individual therapy sessions. Inter‐rater reliability for MBT‐RACS scores was quantified using intraclass correlation coefficients (ICCs). Specifically, a two‐way random‐effects model with absolute agreement and average‐measures ICC (ICC[2,k]) was applied, which accounts for both session and rater variability and assumes raters represent a random sample from a broader population of raters (Koo & Li, 2016). In line with standard practice, both single‐measures ICC (reflecting reliability of individual rater scores) and average‐measures ICC (reflecting reliability of the mean score across raters) are reported. ICC values were computed based on three independent raters per session. To assess robustness under more typical coding conditions, we also recalculated ICCs using a randomly selected subset of two raters per session. ICC values were interpreted according to conventional guidelines: ICC < .50 indicates poor reliability, ICC between .50 and .75 moderate reliability, ICC between .75 and .90 good reliability and ICC > .90 excellent reliability (Koo & Li, 2016). All ICC analyses were performed using SPSS version 30.0.0.

## RESULTS

Descriptive statistics for each MBT‐RACS domain and the overall adherence score are presented separately for group and individual therapy sessions. Table 1 displays means, standard deviations and observed score ranges for each domain.

**TABLE 1:** Descriptive statistics for MBT‐RACS domain scores and overall adherence in group and individual therapy sessions (mean of three raters).

Domain	Group sessions, *N* = 104		Individual sessions, *N* = 22	
	Mean (*SD*)	Range	Mean (*SD*)	Range
Sessional structure	4.15 (.72)	2–6	3.96 (1.32)	1–6
Not‐knowing stance	4.10 (.86)	1–7	3.96 (1.22)	2–7
Mentalizing process	3.97 (.73)	2–6	3.96 (1.31)	1–7
Non‐mentalizing modes	3.86 (.90)	1–7	3.71 (1.54)	1–7
Mentalizing affective narrative	3.96 (.88)	1–6	3.88 (1.35)	1–7
Relational mentalizing	3.56 (.73)	1–5.5	3.24 (1.51)	1–6
Adjusted domain score (including quality how score)	23.60 (3.62)	10–35	22.70 (7.00)	9–40
Final adherence (including quality absence score)	3.94 (.60)	1.67–5.83	3.78 (1.17)	1.50–6.67

Inter‐rater reliability of the MBT‐RACS was strong overall, with most domains demonstrating good to excellent agreement among raters for both group and individual MBT sessions.

### Group MBT sessions

For group sessions (*n* = 104), average‐measures ICCs based on three raters ranged from .74 to .84 across the six rated domains (see Table 2). The highest agreement was observed for the total adherence rating (ICC = .91, 95% CI [.87–.93]). Individual domains, such as identification of non‐mentalizing modes (ICC = .83) and relational mentalizing (ICC = .84), achieved excellent reliability, whereas sessional structure (ICC = .74) and the not‐knowing stance (ICC = .75) displayed somewhat lower, but still good, reliability. Single‐measure ICCs, which reflect expected reliability when rated by a single coder, were more moderate, ranging from .49 to .63 across domains. This suggests individual raters provide reasonable reliability, though averaging scores across multiple raters notably enhances score stability. When recalculating average‐measures ICCs using ratings from two raters—a common practice in research and supervision settings—reliability remained good, ranging from .67 to .78 across individual domains and reaching .84 for the total adherence score.

**TABLE 2:** Inter‐rater reliability of coders across MBT group‐session domains, using intraclass coefficient with a two‐way random‐effects model measuring absolute agreement for both single and average measures.

Group‐session, *N* = 104m item name	Average‐measures (3R) CC (confidence intervals)	Single‐measure (3R) ICC (confidence intervals)	Average‐measures (2R) ICC (confidence intervals)
Mentalizing sessional structure	.74 (.64–.82)	.49 (.38–.60)	.67 (.51–.77)
Not‐knowing stance	.75 (.65–.82)	.50 (.38–.61)	.74 (.62–.82)
Mentalizing process	.82 (.74–.87)	.60 (.49–.69)	.73 (.60–.81)
Non‐mentalizing modes	.83 (.77–.88)	.63 (.53–.71)	.78 (.67–.85)
Mentalizing affective narrative	.80 (.72–.86)	.57 (.46–.67)	.67 (.52–.78)
Relational mentalizing	.84 (.76–.89)	.63 (.52–.74)	.76 (.62–.85)
Quality/absence	.88 (.82–.92)	.71 (.60–.80)	.90 (.83–.94)
Adjusted domain score (including quality how score)	.94 (.82–.96)	.84 (.79–.88)	.90 (.86–.93)
Final adherence (including quality absence score)	.91 (.87–.93)	.76 (.69–.82)	.84 (.77–.89)

Abbreviations: 2R, two raters; 3R, 3 raters.

### Individual MBT sessions

Inter‐rater agreement was even stronger for individual therapy sessions (*n* = 22). Average‐measures ICCs based on three raters ranged from .84 to .95 across the six domains, indicating excellent reliability throughout (see Table 3). The total adherence score yielded an ICC of .98 (95% CI [.96–.99]). Even domains with comparatively lower agreement, such as the not‐knowing stance (ICC = .84), remained within the excellent reliability range. Single‐measure ICCs ranged from .64 to .95, demonstrating robust reliability even at the individual coder level for most domains and the overall adherence score. This suggests that with appropriate training and calibration, single raters can provide acceptable estimates of therapist adherence and competence, though caution should be exercised for domains involving greater interpretive complexity or less frequently observed interventions. Average‐measures ICCs calculated from two raters ranged from .78 to .91, maintaining excellent reliability across all domains. These findings support the feasibility of employing two raters for applied research and clinical supervision settings, particularly when resource constraints prevent triple coding.

**TABLE 3:** Inter‐rater reliability of coders across MBT individual session domains, using intraclass coefficient with a two‐way random‐effects model measuring absolute agreement for both single and average measures.

Individual sessions, *N* = 22 item name	Average‐measures (3R) ICC (confidence intervals)	Single‐measure (3R) ICC (confidence intervals)	Average‐measures (2R) ICC (confidence intervals)
Mentalizing sessional structure	.95 (.88–.98)	.85 (.71–.93)	.91 (.77–.96)
Not‐knowing stance	.84 (.66–.93)	.64 (.40–.82)	.86 (.64–.94)
Mentalizing process	.89 (.76–.96)	.74 (.52–.76)	.78 (.71–.85)
Non‐mentalizing modes	.92 (.82–.97)	.80 (.61–.92)	.82 (.51–.94)
Mentalizing affective narrative	.90 (.79–.96)	.75 (.55–.90)	.84 (.60–.94)
Relational mentalizing	.85 (.53–.96)	.66 (.27–.90)	.84 (.32–.96)
Quality/absence	.87 (.73–.94)	.69 (.48–85)	1.00
Adjusted domain score (including quality how score)	.99 (.97–.99)	.96 (.91–.98)	.98 (.96–.99)
Final adherence (including quality absence score)	.98 (.96–.99)	.95 (.90–.98)	.97 (.94–.99)

Abbreviations: 2R, two raters; 3R, 3 raters.

## DISCUSSION

Our findings demonstrate that all domains of the MBT‐RACS exhibit at least moderate (ICC > .50) to good (ICC > .75) inter‐rater reliability, with several domains surpassing the excellent threshold (ICC > .90). Particularly notable were the high ICCs observed for the total adherence score, indicating that raters rarely disagreed when evaluating overall fidelity. Such robust reliability is highly encouraging, as it supports the dependable use of the MBT‐RACS in determining therapist adherence and competence—essential requirements for effective research assessment and clinical supervision.

The results strongly support the reliability and practical utility of the MBT‐RACS. Ratings were consistently high for both the MBT group and individual sessions, indicating that the scale performs effectively across different therapeutic formats. The consistently good inter‐rater agreement across domains and exceptionally high reliability for total scores underscore the scale's strength. These findings suggest the MBT‐RACS effectively addresses challenges previously encountered with other MBT adherence scales, including lower reliability in scenarios with fewer raters (Karterud et al., 2013). Given the high level of agreement among raters, the MBT‐RACS appears particularly suitable for multi‐site trials and certification contexts, where consistent and accurate ratings of therapist performance are critical.

Notably, our data suggest substantial improvement over earlier MBT adherence instruments. The MBT‐ACS reported overall adherence ICCs of approximately .60 with two raters, whereas our results demonstrate much stronger agreement, with ICCs reaching .84 in group sessions and .95 in individual sessions, firmly within the excellent reliability range. This improvement indicates that the MBT‐RACS's domain‐focused design and new structure have significantly enhanced inter‐rater consistency relative to earlier measures. Domain‐level reliability was uniformly acceptable to excellent, with none of the extremely low reliability previously observed in domains such as ‘pretend mode’ and ‘stop and rewind’, which had ICCs near zero in earlier versions.

The domain ‘identification of non‐mentalizing modes’, which includes techniques previously challenging to rate consistently, now achieved at least moderate reliability, likely due to the reframing and integration of multiple infrequent interventions within a single broader domain. However, while these findings are encouraging, it is important to consider that this improvement in reliability may partly reflect a shift toward more top‐down, domain‐level judgements that aggregate across heterogeneous intervention types. In particular, agreement among raters may be primarily driven by more readily identifiable modes, such as interventions addressing psychic equivalence or teleological thinking, whereas less frequently observed or more subtle techniques, such as recognizing and handling pretend mode or using a ‘stop and rewind’ intervention, may still elicit greater disagreement. This possibility underscores a key trade‐off in the new scoring approach: while broader domains improve overall consistency, they may also mask item‐level variability or rater uncertainty. Future work could explore whether specific subcomponents within this domain are being applied with equal consistency or whether certain intervention types continue to pose reliability challenges even within the improved domain structure.

Despite this potential limitation, the overall reliability of the MBT‐RACS remains strong, including in its integration of adherence and competence into unified domain scores, an approach that was previously separated but shown to be highly correlated (*r* ≈ .93). High inter‐rater reliability was maintained despite this integration, suggesting that simplifying the rating structure did not compromise accuracy or consistency. Indeed, the combined rating approach of frequency and quality of interventions produced one of the highest reliability outcomes, aligning with prior observations by Karterud et al. (2013) regarding overall fidelity assessment. Our findings indicate that overall, the MBT‐RACS efficiently captures fidelity with fewer raters and a streamlined structure, enhancing its practical applicability.

Enhanced reliability of the MBT‐RACS may benefit therapist training, supervision and overall treatment quality in clinical practice by providing clear, consistent benchmarks for evaluating therapist performance. Reliable assessment enables trainers and supervisors to give targeted, actionable feedback based on precise adherence and competence measures, facilitating more efficient skill development. Additionally, consistent ratings ensure that therapists clearly understand the specific behaviours and competencies required to deliver MBT effectively, improving fidelity and therapeutic outcomes. Ultimately, this leads to higher standards of practice, better‐informed supervision and greater confidence among therapists, enhancing overall clinical service delivery.

Future studies, including longitudinal ones, will be essential to establish whether higher adherence and competence scores on the MBT‐RACS predict meaningful clinical outcomes, such as reduced symptom severity or enhanced mentalization capacities in patients. Some early research using the MBT‐ACS suggests a higher adherence and competence score facilitates greater in‐session mentalization (Möller et al., 2017), but Philips et al. (2018) found no impact of adherence and competence on symptom severity of BPD. Demonstrating these predictive relationships with the MBT‐RACS would validate the scale's clinical utility, supporting its broader implementation in both research and practice settings. Additionally, future research should explore the MBT‐RACS's sensitivity to changes in therapist skills over time, particularly throughout structured training or clinical supervision programmes. This would clarify the scale's potential as a responsive tool for tracking therapist development and evaluating the effectiveness of training interventions. Future studies should also examine the relationship between domain scores of the MBT‐RACS and other established therapeutic process measures, such as alliance quality, therapist empathy, reflective functioning, and in‐session attachment behaviour and therapist attachment representations (Talia et al., 2020). Investigating these relationships would strengthen evidence for the scale's construct validity, confirming that higher adherence and competence scores align meaningfully with broader therapeutic processes known to facilitate effective treatment outcomes.

A broader issue within the literature on treatment fidelity is the widespread absence of shared definitions and conceptual frameworks for implementation fidelity (Berry et al., 2025; Giovanazzi et al., 2022). This ambiguity is reflected in inconsistent terminology, with terms such as ‘adherence’, ‘compliance’ and ‘fidelity’ frequently conflated (Giovanazzi et al., 2022; Lemire et al., 2023). Here, adherence is defined as the extent to which specified ingredients or intervention components are delivered as prescribed (Lemire et al., 2023), while fidelity is regarded as an overarching construct encompassing adherence, exposure (dosage), quality and participant responsiveness (Lemire et al., 2023; Páez et al., 2025). This approach aims to enhance definitional clarity and standardization within MBT research, potentially offering a framework for other complex interventions to resolve prevalent conceptual ambiguities in fidelity assessment.

While the results presented in this paper are encouraging, it is important to acknowledge potential limitations. Although our sample size, especially for individual sessions, was relatively modest (*n* = 22), it nonetheless provided robust estimates of reliability. Future research should evaluate the MBT‐RACS in diverse clinical populations and examine its predictive validity regarding treatment outcomes. Continued psychometric validation, including cross‐cultural reliability assessments and exploration of rater training effectiveness, would further strengthen confidence in the MBT‐RACS as an international standard for fidelity assessment in MBT. In light of the international adoption of MBT, future validation studies should be carried out across diverse clinical settings as well as cultural contexts to confirm the scale's international robustness and adaptability, as therapeutic style, patient interaction and interpretations of fidelity may differ culturally. Our study used highly trained and experienced raters, which could introduce potential bias and may limit generalizability to situations involving less intensive training or less experienced coders. Therefore, future research should explicitly test reliability under conditions of more modest rater preparation, for example, abbreviated training workshops for more experienced MBT clinicians or fewer practice tapes, compared to the 1‐day, supervised training and calibration process used in this study. Finally, the relatively smaller sample of individual therapy sessions warrants caution in interpreting those findings; replication with larger samples is recommended to confirm reliability estimates more confidently for individual MBT sessions.

Looking ahead, technological developments, particularly in machine learning (ML) and artificial intelligence (AI), offer significant potential for transforming the coding and assessment of treatment fidelity and adherence to therapeutic models (Malgaroli et al., 2023). Automated analysis of session recordings could streamline the currently labour‐intensive manual rating process, improving the accessibility and cost‐effectiveness of fidelity assessment for routine clinical practice and large‐scale research. Whereas existing methods often rely heavily on human expertise and extensive time commitments, AI‐driven tools promise greater efficiency and objectivity, potentially easing rater burden and enhancing scalability. However, the more interpretive, top‐down structure of the MBT‐RACS may pose challenges for initial AI implementation, as it relies less on discrete utterances and more on global clinical impressions. Nonetheless, this structure could still support supervised learning approaches trained on well‐rated sessions. Such advancements would facilitate more frequent and widespread monitoring of treatment integrity, ultimately promoting higher standards of practice and strengthening the evidence base for interventions like MBT.

## AUTHOR CONTRIBUTIONS

**Karen Yirmiya:** Conceptualization; formal analysis; writing – review and editing; writing – original draft; methodology. **Sophie Marjoribanks:** Formal analysis; writing – review and editing; methodology. **Peter Fonagy:** Conceptualization; supervision; writing – review and editing; writing – original draft. **Anthony Bateman:** Conceptualization; investigation; data curation; supervision; writing – review and editing.

## FUNDING INFORMATION

No funding was received for this study.

## CONFLICT OF INTEREST STATEMENT

The authors declare that they have no conflicts of interest.

## Data Availability

The data supporting the findings of this study are available from the corresponding author upon reasonable request.

## References

[bjc70010-bib-0001] Anna Freud National Centre for Children and Families . (2020). MBT adherence and competence scale . Anna Freud National Centre for Children and Families. https://www.annafreud.org/training/health‐and‐social‐care/mentalization‐based‐treatments‐mbt/mentalization‐based‐treatment‐adults/mbt‐resources/mbt‐adherence‐scale/

[bjc70010-bib-0002] Bate, J. , & Midgley, N. (2020). Mentalization based therapy for children adherence scale (MBT‐CAS) . Yeshiva University; University College London.

[bjc70010-bib-0003] Bateman, A. , & Fonagy, P. (2016). Mentalization‐based treatment for personality disorders: A practical guide. Oxford University Press.

[bjc70010-bib-0004] Bateman, A. , Fonagy, P. , Campbell, C. , Luyten, P. , & Debbané, M. (2023). Cambridge guide to mentalization‐based treatment (MBT). Cambridge University Press.

[bjc70010-bib-0005] Bateman, A. , O'Connell, J. , Lorenzini, N. , Gardner, T. , & Fonagy, P. (2016). A randomised controlled trial of mentalization‐based treatment versus structured clinical management for patients with comorbid borderline personality disorder and antisocial personality disorder. BMC Psychiatry, 16, 1–11. 27577562 10.1186/s12888-016-1000-9 PMC5006360

[bjc70010-bib-0006] Bellg, A. J. , Borrelli, B. , Resnick, B. , Hecht, J. , Minicucci, D. S. , Ory, M. , Ogedegbe, G. , Orwig, D. , Ernst, D. , & Czajkowski, S. (2004). Enhancing treatment fidelity in health behavior change studies: Best practices and recommendations from the NIH behavior change consortium. Health Psychology, 23(5), 443–451. 10.1037/0278-6133.23.5.443 15367063

[bjc70010-bib-0007] Berry, K. , Handerer, F. , Bucci, S. , Penn, G. , Morley, H. , Raphael, J. , Lovell, K. , Price, O. , Edge, D. , Drake, R. J. , & Haddock, G. (2025). Ensuring that psychological interventions are delivered as intended on mental health inpatient wards. The British Journal of Clinical Psychology, 64(2), 371–384. 10.1111/bjc.12510 39467006 PMC12057325

[bjc70010-bib-0008] Bond, G. R. , & Drake, R. E. (2020). Assessing the Fidelity of evidence‐based practices: History and current status of a standardized measurement methodology. Administration and Policy in Mental Health and Mental Health Services Research, 47(6), 874–884. 10.1007/s10488-019-00991-6 31691055

[bjc70010-bib-0009] Byrne, G. , Murphy, S. , & Connon, G. (2020). Mentalization‐based treatments with children and families: A systematic review of the literature. Clinical Child Psychology and Psychiatry, 25(4), 1022–1048. 32493055 10.1177/1359104520920689

[bjc70010-bib-0010] Coşkun, A. , Halfon, S. , Bate, J. , & Midgley, N. (2024). The use of mentalization‐based techniques in online psychodynamic child psychotherapy. Psychotherapy Research, 34(7), 1005–1017. 10.1080/10503307.2023.2245962 37594025

[bjc70010-bib-0011] Folmo, E. J. , Karterud, S. W. , Bremer, K. , Walther, K. L. , Kvarstein, E. H. , & Pedersen, G. A. F. (2017). The design of the MBT‐G adherence and quality scale. Scandinavian Journal of Psychology, 58(4), 341–349. 10.1111/sjop.12375 28718968

[bjc70010-bib-0012] Fonagy, P. , & Bateman, A. W. (2006). Mechanisms of change in mentalization‐based treatment of BPD. Journal of Clinical Psychology, 62(4), 411–430. 16470710 10.1002/jclp.20241

[bjc70010-bib-0013] Fonagy, P. , Gergely, G. , & Jurist, E. L. (2018). Affect regulation, mentalization and the development of the self. Routledge.

[bjc70010-bib-0014] Fonagy, P. , & Luyten, P. (2009). A developmental, mentalization‐based approach to the understanding and treatment of borderline personality disorder. Development and Psychopathology, 21(4), 1355–1381. 10.1017/S0954579409990198 19825272

[bjc70010-bib-0015] Fonagy, P. , Luyten, P. , Allison, E. , & Campbell, C. (2019). Mentalizing, epistemic trust and the phenomenology of psychotherapy. Psychopathology, 52(2), 94–103. 31362289 10.1159/000501526

[bjc70010-bib-0016] Fonagy, P. , Simes, E. , Yirmiya, K. , Wason, J. , Barrett, B. , Frater, A. , Cameron, A. , Butler, S. , Hoare, Z. , & McMurran, M. (2025). Mentalisation‐based treatment for antisocial personality disorder in males convicted of an offence on community probation in England and Wales (mentalization for offending adult males, MOAM): A multicentre, assessor‐blinded, randomised controlled trial. The Lancet Psychiatry, 12(3), 208–219. 39978982 10.1016/S2215-0366(24)00445-0

[bjc70010-bib-0017] Ginsburg, L. R. , Hoben, M. , Easterbrook, A. , Anderson, R. A. , Estabrooks, C. A. , & Norton, P. G. (2021). Fidelity is not easy! Challenges and guidelines for assessing fidelity in complex interventions. Trials, 22(1), 372. 10.1186/s13063-021-05322-5 34051830 PMC8164256

[bjc70010-bib-0018] Giovanazzi, A. , Jones, K. , Carr, R. M. , Fairhurst, C. M. , Backhouse, M. R. , & Adamson, J. A. (2022). Current practice in the measurement and interpretation of intervention adherence in randomised controlled trials: A systematic review. Contemporary Clinical Trials, 118, 106788. 35562000 10.1016/j.cct.2022.106788

[bjc70010-bib-0019] Güvenç, D. , & Halfon, S. (2023). Dynamic relations between mentalization techniques and therapeutic alliance in psychodynamic child therapy: An evidence‐based case study. Psychotherapy (Chicago, Ill.), 60(4), 548–559. 10.1037/pst0000505 37856405

[bjc70010-bib-0020] Hajek Gross, C. , Oehlke, S. M. , Prillinger, K. , Goreis, A. , Plener, P. L. , & Kothgassner, O. D. (2024). Efficacy of mentalization‐based therapy in treating self‐harm: A systematic review and meta‐analysis. Suicide and Life‐Threatening Behavior, 54(2), 317–337. 38279664 10.1111/sltb.13044

[bjc70010-bib-0021] Hauschild, S. , Kasper, L. , Volkert, J. , Sobanski, E. , & Taubner, S. (2023). Mentalization‐based treatment for adolescents with conduct disorder (MBT‐CD): A feasibility study. European Child & Adolescent Psychiatry, 32(12), 2611–2622. 10.1007/s00787-022-02113-4 36434148 PMC9702655

[bjc70010-bib-0022] Karterud, S. , Pedersen, G. , Engen, M. , Johansen, M. S. , Johansson, P. N. , Schlüter, C. , Urnes, Ø. , Wilberg, T. , & Bateman, A. W. (2013). The MBT adherence and competence scale (MBT‐ACS): Development, structure and reliability. Psychotherapy Research, 23(6), 705–717. 22916991 10.1080/10503307.2012.708795

[bjc70010-bib-0023] Koo, T. K. , & Li, M. Y. (2016). A guideline of selecting and reporting intraclass correlation coefficients for reliability research. Journal of Chiropractic Medicine, 15(2), 155–163. 10.1016/j.jcm.2016.02.012 27330520 PMC4913118

[bjc70010-bib-0024] Kvarstein, E. H. , Pedersen, G. , Folmo, E. , Urnes, Ø. , Johansen, M. S. , Hummelen, B. , Wilberg, T. , & Karterud, S. (2019). Mentalization‐based treatment or psychodynamic treatment programmes for patients with borderline personality disorder—The impact of clinical severity. Psychology and Psychotherapy, 92(1), 91–111. 10.1111/papt.12179 29582581 PMC6585931

[bjc70010-bib-0025] Kvarstein, E. H. , Pedersen, G. , Urnes, Ø. , Hummelen, B. , Wilberg, T. , & Karterud, S. (2015). Changing from a traditional psychodynamic treatment programme to mentalization‐based treatment for patients with borderline personality disorder—Does it make a difference? Psychology and Psychotherapy, 88(1), 71–86. 10.1111/papt.12036 25045028 PMC4344810

[bjc70010-bib-0026] Lemire, C. , Rousseau, M. , & Dionne, C. (2023). A comparison of fidelity implementation frameworks used in the field of early intervention. American Journal of Evaluation, 44(2), 236–252. 10.1177/10982140211008978

[bjc70010-bib-0027] Malda‐Castillo, J. , Browne, C. , & Perez‐Algorta, G. (2019). Mentalization‐based treatment and its evidence‐base status: A systematic literature review. Psychology and Psychotherapy, 92(4), 465–498. 10.1111/papt.12195 30091506

[bjc70010-bib-0028] Malgaroli, M. , Hull, T. D. , Zech, J. M. , & Althoff, T. (2023). Natural language processing for mental health interventions: A systematic review and research framework. Translational Psychiatry, 13(1), 309. 10.1038/s41398-023-02592-2 37798296 PMC10556019

[bjc70010-bib-0029] Möller, C. , Karlgren, L. , Sandell, A. , Falkenström, F. , & Philips, B. (2017). Mentalization‐based therapy adherence and competence stimulates in‐session mentalization in psychotherapy for borderline personality disorder with co‐morbid substance dependence. Psychotherapy Research, 27(6), 749–765. 10.1080/10503307.2016.1158433 27093128

[bjc70010-bib-0030] Moncher, F. J. , & Prinz, R. J. (1991). Treatment fidelity in outcome studies. Clinical Psychology Review, 11(3), 247–266. 10.1016/0272-7358(91)90103-2

[bjc70010-bib-0031] Páez, A. , Nunan, D. , McCulloch, P. , & Beard, D. (2025). The influence of intervention fidelity on treatment effect estimates in clinical trials of complex interventions: A metaepidemiological study. Journal of Clinical Epidemiology, 177, 111610. 10.1016/j.jclinepi.2024.111610 39528004

[bjc70010-bib-0032] Philips, B. , Wennberg, P. , Konradsson, P. , & Franck, J. (2018). Mentalization‐based treatment for concurrent borderline personality disorder and substance use disorder: A randomized controlled feasibility study. European Addiction Research, 24(1), 1–8. 10.1159/000485564 29402870 PMC5969093

[bjc70010-bib-0033] Sharp, C. , & Rossouw, T. (2024). Mentalization‐based treatment for adolescents (MBT‐A). Psychodynamic Psychiatry, 52(4), 542–562. 10.1521/pdps.2024.52.4.542 39679703

[bjc70010-bib-0034] Simonsen, S. , Juul, S. , Kongerslev, M. , Bo, S. , Folmo, E. , & Karterud, S. (2019). The mentalization‐based therapy adherence and quality scale (MBT‐AQS): Reliability in a clinical setting. Nordic Psychology, 71(2), 104–115. 10.1080/19012276.2018.1480406

[bjc70010-bib-0035] Talia, A. , Muzi, L. , Lingiardi, V. , & Taubner, S. (2020). How to be a secure base: Therapists' attachment representations and their link to attunement in psychotherapy. Attachment & Human Development, 22(2), 189–206. 10.1080/14616734.2018.1534247 30336734

[bjc70010-bib-0036] Vogt, K. S. , & Norman, P. (2019). Is mentalization‐based therapy effective in treating the symptoms of borderline personality disorder? A systematic review. Psychology and Psychotherapy: Theory, Research and Practice, 92(4), 441–464. 10.1111/papt.12194 PMC6900007 30099834

